# Co-activation of AKT and c-Met triggers rapid hepatocellular carcinoma development via the mTORC1/FASN pathway in mice

**DOI:** 10.1038/srep20484

**Published:** 2016-02-09

**Authors:** Junjie Hu, Li Che, Lei Li, Maria G. Pilo, Antonio Cigliano, Silvia Ribback, Xiaolei Li, Gavinella Latte, Marta Mela, Matthias Evert, Frank Dombrowski, Guohua Zheng, Xin Chen, Diego F. Calvisi

**Affiliations:** 1 School of Pharmacy, Hubei University of Chinese Medicine, Wuhan, Hubei, P.R. China; 2 Department of Bioengineering and Therapeutic Sciences and Liver Center, University of California, San Francisco, CA, USA; 3 Key Laboratory of Carcinogenesis and Translational Research (Ministry of Education), Peking University Cancer Hospital and Institute, Beijing, P. R. China; 4 School of Pharmacy, Tongji Medical College, Huazhong University of Science and Technology, Wuhan, Hubei, P. R. China; 5 Department of Clinical and Experimental Medicine, University of Sassari, Sassari, Italy; 6 Institute of Pathology, University of Greifswald, Greifswald, Germany; 7 Department of Hepatobiliary Surgery, Xijing Hospital, The Fourth Military Medical University, Xi’an, Shaanxi, P.R. China; 8 Institute of Pathology, University of Regensburg, Regensburg, Germany

## Abstract

Activation of the AKT/mTOR cascade and overexpression of c-Met have been implicated in the development of human hepatocellular carcinoma (HCC). To elucidate the functional crosstalk between the two pathways, we generated a model characterized by the combined expression of activated AKT and c-Met in the mouse liver. Co-expression of AKT and c-Met triggered rapid liver tumor development and mice required to be euthanized within 8 weeks after hydrodynamic injection. At the molecular level, liver tumors induced by AKT/c-Met display activation of AKT/mTOR and Ras/MAPK cascades as well as increased lipogenesis and glycolysis. Since a remarkable lipogenic phenotype characterizes liver lesions from AKT/c-Met mice, we determined the requirement of lipogenesis in AKT/c-Met driven hepatocarcinogenesis using conditional Fatty Acid Synthase (FASN) knockout mice. Of note, hepatocarcinogenesis induced by AKT/c-Met was fully inhibited by FASN ablation. In human HCC samples, coordinated expression of FASN, activated AKT, and c-Met proteins was detected in a subgroup of biologically aggressive tumors. Altogether, our study demonstrates that co-activation of AKT and c-Met induces HCC development that depends on the mTORC1/FASN pathway. Suppression of mTORC1 and/or FASN might be highly detrimental for the growth of human HCC subsets characterized by concomitant induction of the AKT and c-Met cascades.

Hepatocellular carcinoma (HCC) is the most common histologic type of primary liver cancer and the third leading cause of cancer-related death worldwide^1^,^2^,^3^. HCC is characterized by fast infiltrating growth, early intrahepatic metastases, high-grade malignancy, and poor prognosis^1^,^2^,^3^. The treatment options for HCC are very limited when the tumor is not resectable^1^,^2^,^3^,^4^,^5^. Thus, the elucidation of the molecular pathogenesis of HCC is of prime importance to develop novel therapeutic strategies against this deadly disease.

The PI3K/AKT/mTOR cascade is one of the critical signaling pathways implicated in hepatocarcinogenesis^6^,^7^. In this cascade, activation of phosphoinositide-3 kinase (PI3K) leads to phosphorylation and activation of v-akt murine thymoma viral oncogene homolog (AKT), a serine/threonine kinase, which in turn induces its major downstream effector, the mTOR complex 1 (mTORC1)^6^,^7^. The major downstream regulators of mTORC1 are the 4-EBP1/eIF4E and p70S6K/RPS6 cascades^6^,^7^. In particular, 4-EBP1/eIF4E controls cap-dependent translation, whereas p70S6K/RPS6 is the major regulator of cell metabolism and proliferation^6^,^7^.

The *c-Met* gene encodes the receptor tyrosine kinase for hepatocyte growth factor (HGF) and scatter factor (SF)^8^,^9^,^10^,^11^. c-Met has been shown to be involved in a variety of cellular processes, including cell proliferation, survival, malignant transformation, and metastasis^8^,^9^,^10^,^11^. In the canonical HGF/c-Met pathway, HGF binds to c-Met, leading to homodimerization and autophosphorylation of the latter protein, with consequent activation of the mitogen-activated protein kinase (MAPK) and phosphoinositide-3 kinase (PI3K) pathways^8^,^9^,^10^,^11^. In human HCC, c-Met is often overexpressed, and c-Met levels are associated with tumor biological aggressiveness^9^,^10^,^11^,^12^. Due to the aforementioned features, c-Met might represent a valid target for HCC treatment^9^,^10^,^11^,^12^.

Metabolic reprogramming, including aberrant glucose, glutamine, nucleotide, and lipid metabolism is considered a cancer hallmark^13^. In particular, increased *de novo* fatty acid synthesis is an important feature of malignant transformation and tumor progression^14^,^15^. Importantly, a body of epidemiological evidence has demonstrated that metabolic syndrome and its hepatic manifestations, including non-alcoholic steatohepatitis (NASH) and non-alcoholic fatty liver disease (NAFLD), are important risk factors for HCC^16^,^17^, thus linking deregulated lipid metabolism to liver carcinogenesis. Fatty Acid Synthase (FASN), the key *de novo* lipogenic enzyme catalyzing the synthesis of palmitate from acetyl-CoA and malonyl-CoA, has been found to be upregulated in multiple cancer types, including HCC^14^,^15^,^18^. At the molecular level, mTORC1 is considered to be the major regulator of FASN-mediated fatty acid synthesis during cancer development^19^. Accordingly, our previous findings indicate that activated AKT/mTOR signaling in the mouse liver upregulates FASN expression and promotes lipogenesis, leading to hepatic steatosis^19^,^20^. However, the precise mechanisms whereby FASN and *de novo* lipogenesis contribute to tumorigenesis remain to be better defined.

While increasing evidence indicates that AKT^6^,^7^,^21^ and c-Met^9^,^10^,^11^,^12^ are frequently overexpressed or activated in HCCs, the functional crosstalk between the two pathways and the mechanisms whereby AKT and c-Met cooperate in liver cancer remain obscure. In the present study, we stably expressed AKT and/or c-Met protooncogenes, along with the sleeping beauty transposase (SB), in the mouse liver using hydrodynamic transfection. The downstream pathways mediating AKT/c-Met driven hepatocarcinogenesis have been investigated using *in vivo* conditional knockout mice or shRNA silencing. Our data demonstrate that activation of AKT cooperates with c-Met to promote rapid HCC development in mice via the mTORC1/FASN pathway.

## Results

### Overexpression of activated AKT and c-Met induces rapid liver tumor development in mice

**Figure 1:**
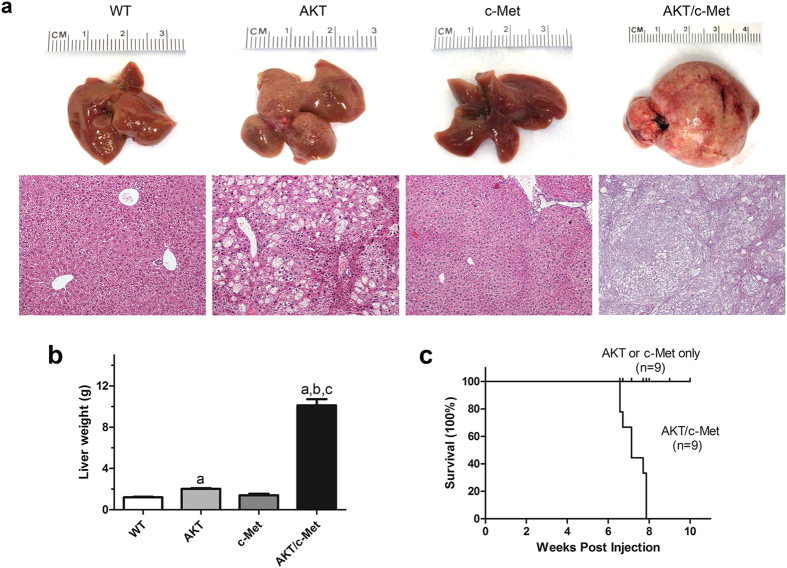
Co-activation of AKT and c-Met promotes liver tumor development in mice. (**a**) Gross images and HE staining of wild-type (WT), AKT, c-Met, and AKT/c-Met mouse livers at 15 weeks of age (eight weeks post hydrodynamic injection in AKT, c-Met, and AKT/c-Met mice). (**b**) Liver weight of wild-type (WT), AKT, c-Met, and AKT/c-Met mice. Tukey-Kramer’s test: *P* at least <0.001; *a*, versus WT livers; *b*, versus AKT HCCS; *c*, versus c-Met livers. (**c**) Survival curve of AKT or c-Met, and AKT/c-Met injected mice. Abbreviation: HE, hematoxylin and eosin staining. Original magnification: 200× in WT, AKT, and c-Met; 100× in AKT/c-Met.

To determine whether c-Met cooperates with activated AKT to induce hepatocarcinogenesis *in vivo*, we stably expressed pT3-EF1α-HA-myr-AKT1 and/or pT3-EF1α-V5-c-Met, along with the sleeping beauty transposase (SB), in the mouse liver using hydrodynamic transfection. As we described previously^19^,^20^, overexpression of AKT alone induced hepatic steatosis and proliferation, leading to HCC development after 24 weeks post hydrodynamic injection. Overexpression of c-Met alone did not result in any liver anomaly in mice up to 12 weeks post injection, but eventually led to dysplastic foci formation over long term, as previously reported^22^,^23^. In striking contrast, co-expression of AKT and c-Met triggered rapid liver tumor development in mice, and all mice developed lethal burden of liver tumor within 6 to 8 weeks post-injection (Fig. 1).

**Figure 2:**
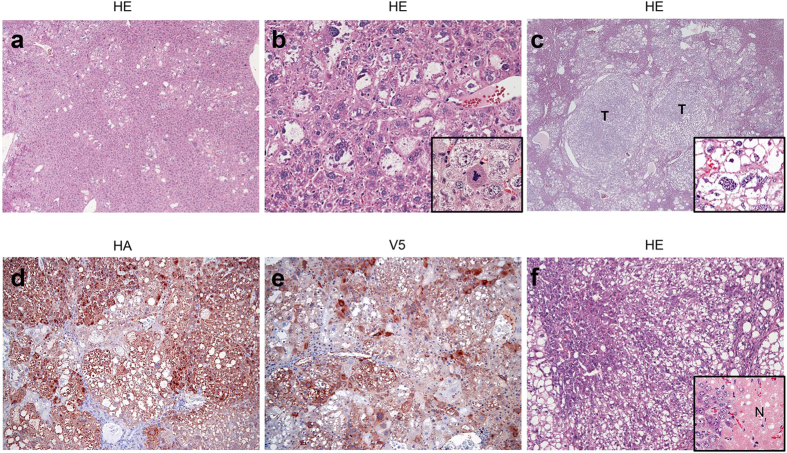
Time course of tumor development in mice co-expressing AKT and c-Met protooncogenes. (**a**) Four weeks after hydrodynamic transfection, AKT/c-Met livers exhibited numerous preneoplastic hepatocytes with a clear cell phenotype due to elevated lipid storage. (**b**) Some of these premalignant hepatocytes were proliferating, as shown by a mitotic figure (**inset**). (**c**) Six weeks after hydrodynamic transfection, numerous hepatocellular tumors (**T**) consisting mainly of lipid-rich cells (**inset**) occupied most of the liver parenchyma of AKT/c-Met mice. These tumors were homogeneously immunoreactive for HA-tagged myr-AKT1 (**d**) and V5-tagged human c-Met (**e**), implying their origin from doubly-transfected cells. (**f**) Eight weeks after injection, hepatocellular tumors occupied almost completely the liver parenchyma of AKT/c-Met mice, and exhibited additional signs of malignancy, including areas of necrosis(**N**) and loss of lipid content by tumor cells (**inset**). Abbreviation: HE, hematoxylin and eosin staining. Original magnification: 200× in (**a**,**b**,**f**); 100× in (**c**,**d**,**e**).

Histopathological analysis was performed to assess the time course of tumor development in AKT/c-Met mice. Four weeks after hydrodynamic transfection, livers of AKT/c-Met mice (n = 3) displayed numerous preneoplastic hepatocytes that contained elevated amounts of glycogen (as assessed by positive PAS reaction; not shown) and lipids (Fig. 2a,b), thus resembling preneoplastic hepatocytes developed in AKT-overexpressing mice^19^,^20^. Approximately 30–40% of the liver volume consisted of preneoplastic lesions, but no tumors were detected (Fig. 2a,b). Six weeks after injection, numerous hepatocellular tumors consisting mainly of lipid-rich cells were found in AKT/c-Met mice (n = 3; Fig. 2c). AKT/c-Met mice (n = 9) developed lethal burden of liver tumor and were required to be euthanized 8 weeks post injection. Hepatocellular tumors further progressed in size, occupying almost completely the liver parenchyma (Fig. 2f). At this time point, AKT/c-Met tumors displayed additional signs of malignancy and aggressiveness, such as confluent areas of necrosis and increase in cytologic atypia as well as a loss of lipid content (Fig. 2f). Indeed, tumors were of mixed cell type (admixture of clear, lipid-rich and lipid-poor, basophilic cells), with basophilic cells being smaller than lipid-rich hepatocytes and sometimes showing an oval-cell phenotype (Fig. 2f). Of note, different from AKT mice, no cholangiocellular lesions were detected in AKT/c-Met mice. To confirm that the liver tumors were indeed induced by the ectopically injected oncogenes, we performed immunohistochemistry (IHC) in preneoplastic and neoplastic lesions from AKT/c-Met mice using an anti-HA and anti-V5-tag antibody, which labelled the ectopically injected AKT and c-Met, respectively. As expected, strong immunolabeling for HA-tag and V5-tag, co-localizing in preneoplastic and neoplastic lesions from AKT/c-Met mice, was detected (Fig. 2d,e; Supplementary Figure 1).

### AKT/c-Met co-expression promotes activation of the AKT/mTOR and Ras/MAPK pathways in the mouse liver

**Figure 3:**
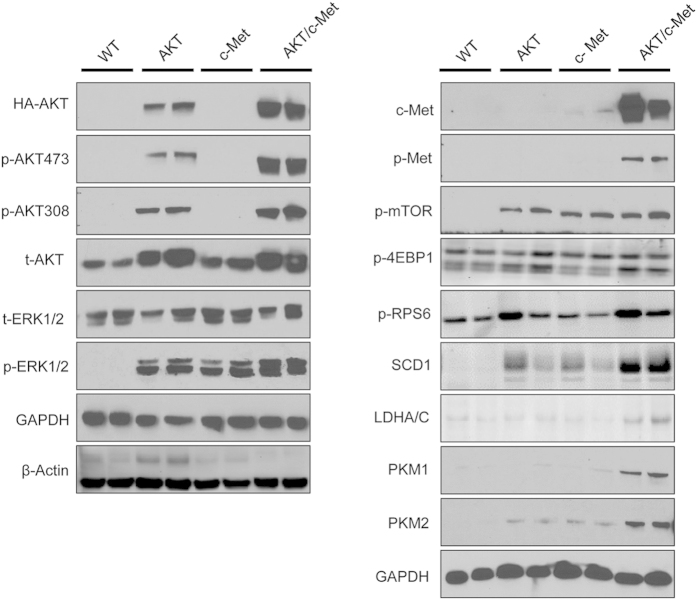
Levels of activation of AKT/mTOR and Ras/MAPK pathways in wild-type (WT), AKT, c-Met, and AKT/c-Met mice. Three to five samples per each group were used for the Western blot analysis, and representative images are shown. GAPDH and β-Actin were used as loading controls.

To elucidate the molecular mechanisms underlying hepatocarcinogenesis in AKT/c-Met mice, we assessed the activation of AKT/mTOR and Ras/MAPK pathways in these mice by Western blotting (Fig. 3). Levels of total AKT were equivalent in liver lesions from AKT and AKT/c-Met mice and higher than in wild-type and c-Met mice, whereas expression of activated/phosphorylated AKT, including p-AKT(T308) and p-AKT(S473), was highest in AKT/c-Met tumors (Fig. 3). Activation of the Ras/MAPK cascade, as indicated by p-ERK1/2 levels, was also highest in AKT/c-Met liver tumors (Fig. 3).

Next, we examined the levels of the major downstream effector cascade of AKT, namely the mTOR pathway. While levels of activated/phosphorylated mTOR and ribosomal protein S6 (RPS6) as well as inactivated/phosphorylated 4E binding protein one (4EBP1) were equivalent in AKT, c-Met, and AKT/c-Met livers, upregulation of mTORC1 downstream effectors involved in *de novo* lipogenesis (stearoyl-CoA desaturase 1 or SCD1) and glycolysis (pyruvate kinase M1 or PKM1, PKM2, and lactate dehydrogenase A/C or LDHA/C) was most pronounced in AKT/c-Met samples (Fig. 3).

Altogether, these results indicate that activation of AKT/mTOR and Ras/MAPK cascades is a molecular feature of AKT/c-Met driven hepatocarcinogenesis.

### Aggressive hepatocarcinogenesis induced by AKT and c-Met co-expression is abolished by suppression of mTORC1/FASN pathway in mice

**Figure 4:**
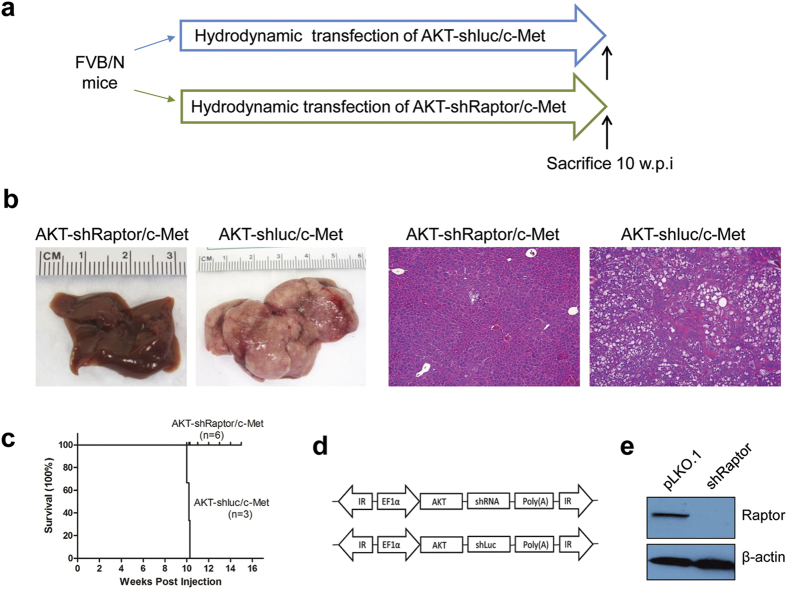
A functional mTORC1 is required for AKT/Met driven tumor development. (**a**) Study design. (**b**) Gross images (left panels) and HE staining (right panels) of AKT-shluc/c-Met and AKT-shRaptor/c-Met livers. (**c**) Survival curve of AKT-shluc/c-Met and AKT-shRaptor/c-Met injected mice. (**d**) Constructs used in the study for the hydrodynamic transfection. (**e**) Western blot analysis confirming Raptor silencing by shRaptor in the AKT/Ras cell line. Abbreviation: HE, hematoxylin and eosin staining. Original magnification: 100× in (**b**).

Next, since lipogenesis and glycolysis are primarily regulated in the liver by mTORC1, we determined whether an intact mTORC1 axis is needed for AKT/c-Met hepatocarcinogenesis in mice. To achieve this goal, we applied miR-30 based shRNA to silence Raptor^24^, the unique subunit of mTORC1 complex *in vivo* (Fig. 4). A previously described shRaptor sequence that showed efficient silencing of mouse Raptor was selected and used for the experiments^25^. We further validated the efficiency of shRaptor in the AKT/Ras mouse liver tumor cell line (Fig. 4e), which has been previously described^20^. The shRaptor sequence was cloned downstream of AKT in the pT3-EF1a vector (AKT-shRaptor). As a control, shRNA against Renilla Luciferase was also cloned into the pT3-EF1a-AKT plasmid (AKT-shLuc) (Fig. 4d). AKT-shRaptor or AKT-shLuc was hydrodynamically injected into mice together with c-Met (Fig. 4a). Consistent with results obtained in AKT/c-Met mice, all AKT-shLuc/c-Met injected mice developed lethal burden of liver tumors and needed to be euthanized by 10 weeks post injection. In striking contrast, liver tissues from AKT-shRaptor/c-Met injected mice appeared to be completely normal at the same time point (Fig. 4b,c). Thus, the present data indicate that AKT/c-Met driven hepatocarcinogenesis depends on functional mTORC1.

**Figure 5:**
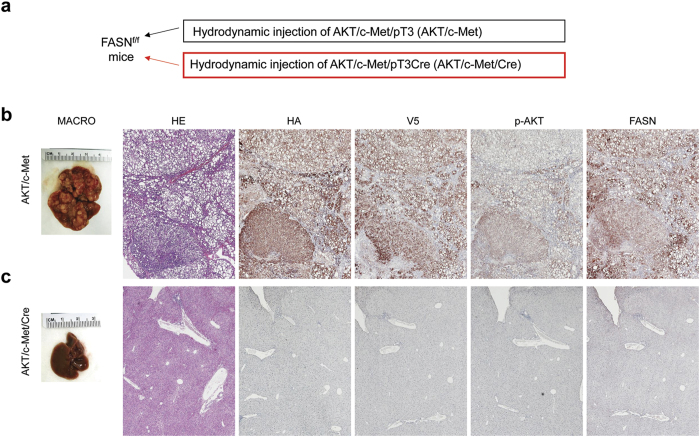
AKT/c-Met hepatocarcinogenesis is completely abolished by depletion of FASN in mice. (**a**) Scheme of the experiment. (**b**) Simultaneous overexpression of the *myr-AKT1* and *c-Met* proto-oncogenes in *FASN*^*fl/fl*^ mice retaining an intact *FASN* gene (indicated in the figure as AKT/c-Met) triggered rapid hepatocarcinogenesis with lethal burden of hepatocellular tumors by 8 weeks post hydrodynamic injection. These tumors as well as preneoplastic lesions were homogeneously immunoreactive for HA-tagged myr-AKT1 (HA) and V5-Met (c-Met), implying their origin from the transfected cells. In addition, preneoplastic and neoplastic lesions exhibited strong immunolabeling for phosphorylated/activated AKT (p-AKT) and FASN. (**c**) Of note, Cre-mediated depletion of *FASN* gene in *FASN*^*fl/fl*^ mice injected with *myr-AKT1* and *c-Met* (here indicated as AKT/c-Met/Cre) completely suppressed tumor development, with no altered cells detectable. This was accompanied by very low/absent immunoreactivity for HA, p-AKT, and FASN in AKT/Met/Cre livers. Original magnification: 40× in (**b**,**c**).

To further investigate the importance of metabolic components regulated by mTORC1 in AKT/c-Met hepatocarcinogenesis, we determined the effect of disrupting *de novo* lipogenesis in these mice. For this purpose, we inactivated FASN, the major player in aberrant lipid biosynthesis^14^,^15^, using conditional *FASN* knockout mice (*FASN*^*fl/fl*^ mice). Thus, two approaches were applied. In the first approach, AKT, c-Met and Cre plasmids were co-injected into *FASN*^*fl/fl*^ mice (AKT/c-Met/Cre; n = 3), thus allowing the simultaneous expression of AKT and c-Met oncogenes while deleting *FASN* in a subset of mouse hepatocytes. As a control, AKT, c-Met and pT3-EF1α (empty vector) were co-injected into *FASN*^*fl/fl*^ mice (AKT/c-Met/pT3, n = 3) (Fig. 5a). We found that all AKT/c-Met/pT3 mice developed liver tumors by 8 weeks post injection (Fig. 5b), whereas no macroscopic or histopathological alterations were detected in the livers of AKT/c-Met/Cre mice up to 15 weeks post hydrodynamic injection (Fig. 5c). At the molecular level, AKT/c-Met/Cre mice displayed very faint or no immunoreactivity for AKT (HA) and c-Met (V5) tags as well as for FASN, p-AKT, SCD1, LDHA/C, p-RPS6, p-4EBP1, and p-ERK1/2 (Fig. 5c and Supplementary Figure 2). The lack of expression of ectopically injected AKT and c-Met in AKT/c-Met/Cre mice might be the consequence of apoptosis of the oncogene-expressing hepatocytes depleted of FASN.

To complement this study, we hydrodynamically transfected AKT/c-Met into liver specific *FASN* knockout mice (*AlbCre;FASN*^*fl/fl*^) mice by crossing *AlbCre* mice with *FASN*^*fl/fl*^ mice as well as control *FASN*^*fl/fl*^ littermates (Supplementary Figure 3). Once again, while all AKT/c-Met injected *FASN*^*fl/fl*^ mice (n = 5) developed high burden of liver tumors by 8 weeks post injection and required to be euthanized, none of the AKT/c-Met injected *AlbCre;FASN*^*fl/fl*^ mice (n = 5) showed any sign of palpable abdominal mass. When harvested 20 weeks post injection, liver tissues from the latter mice appeared to be normal and no preneoplastic or neoplastic lesions were identified (Supplementary Figure 3).

We next determined whether exogenous administration of lipids was able to compensate the loss of *FASN* in AKT/c-Met mice. For this purpose, AKT/c-Met/Cre injected *FASN*^*fl/fl*^ mice (n = 5) were fed a high fat diet (HFD), starting from the second day after hydrodynamic injection, for 10 weeks (Supplementary Figure 4). Of note, HFD administration did not compensate loss of FASN in AKT/c-Met/Cre injected *FASN*^*fl/fl*^ mice (Supplementary Figure 4). Indeed, livers of AKT/c-Met/Cre injected *FASN*^*fl/fl*^ mice appeared macroscopically pale and showed extensive lipid accumulation in hepatocytes 10 weeks post-injection, but did not show any sign of malignant transformation (Supplementary Figure 4).

### FASN post-transcriptionally regulates the levels of c-Met in human hepatoma cell lines

**Figure 6:**
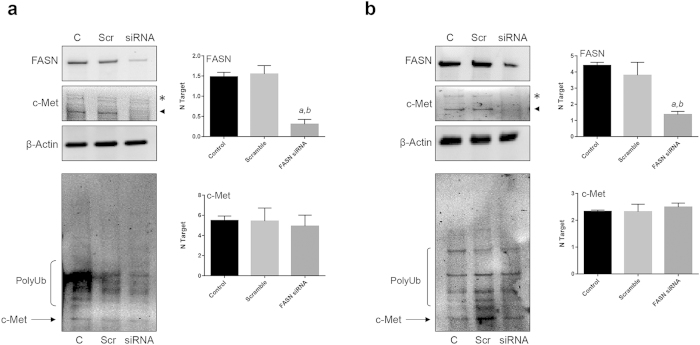
Suppression of FASN post-transcriptionally downregulates c-Met in human hepatoma cell lines. (**a**) In HLF cells, FASN silencing via specific siRNA resulted in the downregulation of c-Met, as detected by Western blot analysis (**a**; left panel). Equivalent results were obtained in HepG2 cells (**b**; left panel). Of note, silencing of FASN did not affect the mRNA levels of c-Met in the two cell lines, as detected by quantitative real-time RT-PCR (**a,b**; right panels). Similarly, no effect of FASN silencing on the levels of ubiquitinylated c-Met was detected (**a,b**; low panels). For Western blot analysis, β-Actin was used as a loading control. Asterisk and arrowhead indicate the pre-form and the cleaved/activated form of c-Met, respectively. For real-time RT-PCR, N target (NT) = 2^−ΔCt^, wherein ΔCt value of each sample was calculated by subtracting the average Ct value of the target gene from the average Ct value of the β-Actin gene. Each bar represent mean ± SD of three independent experiments conducted in triplicate in each cell line. Tukey-Kramer’s test: *P* at least < 0.001; *a*, versus control; *b*, versus scramble siRNA (scramble). Abbreviations: C, control (untreated); Scr, scramble; PolyUb, poly-ubiquitinylated.

Since previous studies demonstrated that inhibition of FASN suppresses c-Met expression in lymphoma cells^26^, we investigated whether the same mechanism may contribute to the suppression of liver tumor development AKT/c-Met mice when FASN is deleted. For this purpose, *FASN* expression was modulated in human hepatoma cell lines and protein levels of c-Met were assessed (Fig. 6). Silencing of *FASN* via specific siRNA resulted in a pronounced downregulation of c-Met protein in HLF and HepG2 cell lines (Fig. 6a,b). To ascertain whether FASN inhibition affects c-Met transcription, thus accounting for the loss of c-Met protein, we performed real-time quantitative reverse-transcription PCR on RNA prepared from HLF and HepG2 cells untreated and subjected to scramble and FASN siRNA. Intriguingly, we found that mRNA levels of *c-Met* gene were unmodified by *FASN* silencing when compared with those from untreated and scramble-treated liver tumor cells (Fig. 6a,b), indicating that regulation of c-Met by FASN occurs at the post-transcriptional level. Next, we assessed whether cap-dependent translation is responsible for the regulation of c-Met levels in HCC cells. For this purpose, the HepG2 cell line was subjected to the treatment with the cap-dependent translation inhibitor, 4EGI-1 (Supplementary Figure 5). Administration of 4EGI-1 did not result in a decrease but rather in an upregulation of c-Met levels (Supplementary Figure 5a). The latter findings seem to exclude that c-Met is positively regulated by cap-dependent translation in HepG2 cells. To further investigate the possible mechanism(s) responsible for c-Met downregulation in FASN-depleted cells, we compared the rate of c-Met loss on treatment with the *de novo* protein synthesis inhibitor, cycloheximide (CHX), either alone or in combination with the FASN inhibitor, C75. Noticeably, loss of c-Met protein was equivalent in CHX- and C75-treated HepG2 cells 48h after the treatment started (Supplementary Figure 5b), whereas combined treatment with C75 and CHX did not result in a synergistic or additive effect on reducing c-Met protein stability (Supplementary Figure 5b). These results suggest that FASN inhibition might reduce c-Met protein stability in HepG2 cells. Furthermore, we determined whether protein degradation via the proteasome system is involved in c-Met downregulation in HCC cells. However, no increase, but rather decrease, in the ubiquitinylated levels of c-Met was detected following *FASN* silencing (Fig. 6a,b) in both HLF and HepG2 cells, thus excluding that FASN regulates c-Met levels via the proteasome system.

Altogether, our data indicate that FASN might contribute to preserve c-Met protein stability in HCC cells.

### Combined inhibition of AKT and c-Met is highly detrimental for the *in vitro* growth of human HCC cell lines

Next, we determined the importance of AKT and c-Met cascades in HCC cell lines. To select the cell lines that might benefit from AKT and c-Met inhibitory treatment, the levels of total and phosphorylated/activated AKT and c-Met proteins were evaluated in HuH6, HuH7, HLE, SK-Hep1, HLF, and HepG2 cell lines (Supplementary Figure 6). Due to elevated levels of activated/phosphorylated AKT and c-Met, the HLF and HLE cell lines were chosen and subjected to the treatment with the AKT inhibitor, MK2206, either alone or in association with the c-Met inhibitor, EMD1214063. Both inhibitors alone were able to decrease proliferation and to augment apoptosis in HLF and HLE cells. An important, additive effect on reduction on proliferation and induction of apoptosis was observed when the two drugs were administered combinatorially (Supplementary Figure 7), indicating that simultaneous targeting of both cascades is detrimental for the growth of HCC cell lines *in vitro*. Notably, treatment with MK2206 resulted also in downregulation of c-Met phosphorylation/activation (Supplementary Figure 6), thus implying a crosstalk between the two oncoproteins in human HCC cells.

### Overexpression of FASN, activated AKT, and c-Met in human HCC specimens

**Figure 7:**
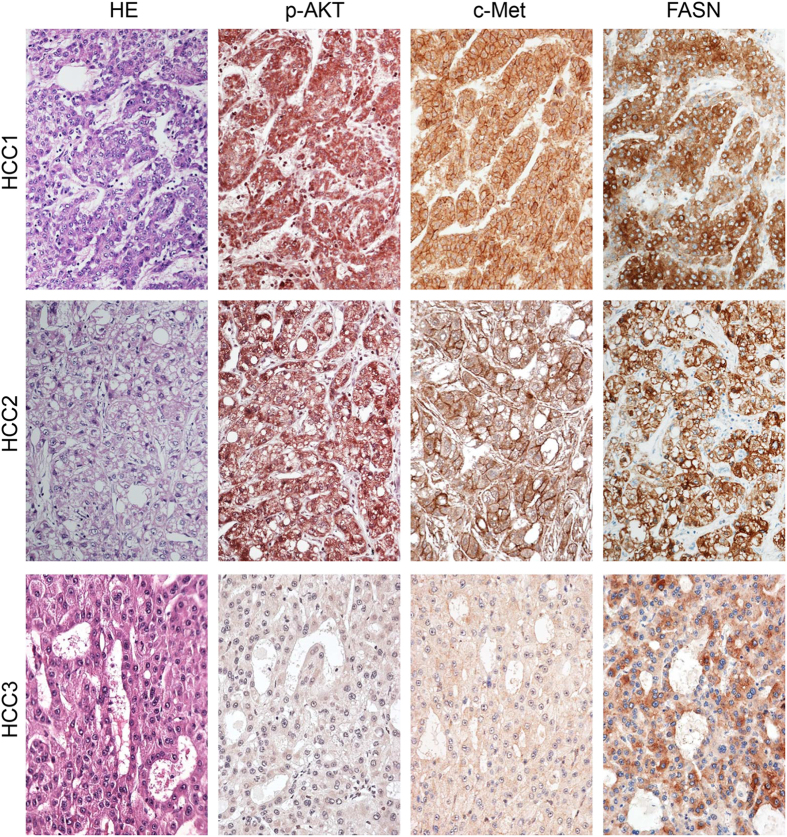
Immunohistochemical patterns of FASN, activated/phosphorylated AKT, and c-Met in human hepatocellular carcinoma (HCC) specimens. Upper panel and middle panel: two HCC specimens (indicated as HCC1 and HCC2) showing strong immunolabeling for FASN, activated/phosphorylated AKT (p-AKT), and c-Met. Lower panel: HCC specimen (indicated as HCC3) with low/absent immunoreactivity for p-AKT and c-Met, and patchy/moderate immunolabeling for FASN. Abbreviation: HE, hematoxylin and eosin staining. Original magnification: 200×.

Finally, given the strong anti-neoplastic effect induced by FASN depletion in AKT/c-Met mouse livers and the molecular mechanisms involved, we assessed the frequency of HCC patients who might eventually benefit from FASN inhibition. For this purpose, levels of FASN, phosphorylated/activated (p-)AKT at serine 473, and c-Met were determined in a collection of human HCC specimens (n = 94; Supplementary Table 1) by immunohistochemistry (Fig. 7). Higher immunoreactivity for FASN, p-AKT, and c-Met proteins was found in 90.4%, 59.6%, and 28.7% of HCC specimens, respectively, when compared with surrounding non-tumorous liver tissue. Importantly, all HCC specimens showing p-AKT and c-Met overexpression also exhibited elevated levels of FASN. Also, 24 of 27 (88.9%) specimens with elevated c-Met concomitantly exhibited induction of p-AKT. In addition, 31 of 50 (66.03%; P < 0.02), and 19 of 27 (70.3%; P < 0.003) of liver tumors displaying induction of p-AKT and c-Met, respectively, belonged to the HCC subset with poorer outcome, linking the overexpression of these proteins to a dismal prognosis of HCC patients. No association between the staining patterns of FASN, p-AKT, and c-Met and other clinicopathological features of the patients, including etiology, presence of cirrhosis, α-fetoprotein levels, and tumor grading was detected.

In summary, the present findings indicate that concomitant induction of FASN, p-AKT, and c-Met proteins characterizes a biologically aggressive subset of human HCC.

## Discussion

Mounting evidence underlines the role of AKT and c-Met proto-oncogenes in human HCC^6^,^7^,^8^,^9^,^10^,^11^,^12^,^21^. However, whether AKT and c-Met functionally cooperate in liver cancer remains poorly delineated. In the present study, we have addressed this issue for the first time in mice. Our results show indeed that concomitant overexpression of AKT and c-Met in the mouse liver results in a synergistic activity of the two proto-oncogenes, leading to rapid tumor development. In accordance with the mouse data, we have found that combined suppression of AKT and c-Met signaling cascades is highly detrimental for the *in vitro* growth of human HCC cell lines. Importantly, suppression of AKT activity by its specific inhibitor, MK2206, resulted in the downregulation of activated c-Met in both HLE and HLF cells. Although the mechanisms whereby AKT regulates c-Met activity require additional investigation, the present data uncover a previously unrevealed crosstalk between AKT and c-Met proteins in HCC cells.

Histologically, preneoplastic and neoplastic lesions developed in AKT/c-Met mice, mainly consisting of lipid-rich cells, closely resemble those from mice overexpressing AKT alone^19^,^20^. However, different from AKT mice, in which liver lesions with hepatocytic, ductular, and mixed differentiation developed^19^,^20^, AKT/c-Met mice exhibited only lesions with hepatocytic features. Thus, these data imply that overexpression of c-Met promotes the development of liver lesions characterized by a commitment toward the hepatocyte lineage. Although the mechanisms whereby c-Met drives development of liver tumors with hepatocytic differentiation remain to be elucidated, our present data are in agreement with a recent report using liver cell lines in which the expression of c-Met and epidermal growth factor receptor (EGFR) has been modulated^27^. In the latter study, the authors showed in fact that c-Met is a strong inducer of hepatocyte differentiation, whereas EGFR promotes cholangiocyte specification while concomitantly suppressing hepatocyte commitment via NOTCH-dependent mechanisms^27^.

At the molecular level, we found that simultaneous overexpression of AKT and c-Met in the liver triggers the sustained activation of the AKT/mTOR and Ras/MAPK cascades. Of note, AKT/c-Met lesions displayed the selective induction of mTOR targets involved in glycolysis and *de novo* lipogenesis, whereas the levels of other canonical effectors of this pathway, such as p-RPS6 and p-4EBP1, were not upregulated when compared with AKT corresponding lesions. Together with the metabolic effects resulting from FASN suppression, however, we cannot exclude that FASN plays also additional roles on AKT/c-Met cells. For instance, FASN depletion either *in vivo* or *in vitro* resulted in the downregulation of c-Met protein, with no changes in c-Met mRNA levels, in accordance with previous data in human breast, prostate, and lung cancer cell lines^28^,^29^. In addition, our data speak against a major role played by the proteasome system in the regulation of c-Met by FASN, as ubiquitination of c-Met was not increased following FASN silencing in hepatoma cell lines. Of note, the negative regulation of c-Met protein levels by FASN independent of the proteasome system has been previously reported in the DU145 prostate cancer cell line following the treatment with the FASN inhibitor, luteolin^28^, further indicating that downregulation of c-Met occurs via mechanisms that are ubiquitin-independent in cancer. Nonetheless, our present data suggest that FASN might be implicated in the regulation of c-Met protein stability, as the loss of c-Met protein in HepG2 cells was equivalent following the treatment with the FASN inhibitor C75 and the protein synthesis inhibitor CHX, whereas the two inhibitors did not act synergistically to downregulate c-Met when used in combination. Concerning the precise mechanisms whereby FASN regulates c-Met stability and activity, it has been recently hypothesized that FASN activity maintains lipid rafts, which may help to stabilize the levels of c-Met^28^. Lipid rafts are plasma membrane regions that regulate cellular signaling, at least partly through the compartmentalization of growth factor receptors^30^,^31^. Since it has been shown that the active form of c-Met resides in lipid rafts^32^, it is possible that disruption of lipid rafts following FASN suppression might trigger the inhibition of c-Met signaling. However, other mechanisms might also play an important role in FASN-mediated control over c-Met levels. For instance, FASN may regulate c-Met levels via microRNA modulation. In accordance with this hypothesis, recent studies showed that fatty acids are important regulators of microRNAs in the liver^33^. Thus, it would be important to further test whether FASN influences the activity of microRNAs that regulate the expression of c-Met in HCC.

Importantly, the current study expands the observation that FASN and its mediated lipogenesis are required for AKT driven carcinogenesis. Our group^34^ and others^35^ have previously reported that AKT-overexpressing cells are incapable of survival and proliferation *in vitro* when *de novo* fatty acid synthesis is inhibited. It is worthwhile remarking that hepatocytes overexpressing *AKT* still rely on FASN when *c-Met* is co-expressed. Indeed, despite the strong acceleration of hepatocarcinogenesis driven by co-transfection of *AKT* and *c-Met* protooncogenes in the mouse liver, *AKT/Met*-overexpressing cells are still dependent on the presence of FASN to exert their oncogenic potential. Furthermore, we found that *AKT/Met* dependent hepatocarcinogenesis is not rescued by dietary fatty acids supplementation in AKT/Met mice depleted of FASN, indicating that AKT/Met cells are unable to compensate the inhibition of *de novo* fatty acid synthesis with exogenous fatty acid uptake. Thus, the present findings indicate that *c-Met* upregulation hastens tumor development in *AKT*-injected hepatocytes albeit without rendering these cells resistant to *FASN* depletion.

Nonetheless, the impact of FASN inhibition might be not limited to liver tumors overexpressing *AKT* and *c-Met*. In accordance with the latter hypothesis, we have recently found that ablation of *FASN* strongly delays c-Myc induced liver tumor development in the mouse (Che L, unpublished results). Thus, our findings together support the hypothesis that increased *de novo* lipogenesis is a key metabolic feature of hepatocarcinogenesis, presumably not limited to AKT/c-Met overexpressing tumors. As a lipogenic phenotype characterizes preneoplastic and neoplastic murine liver lesions as well as human HCC and predisposing conditions (NASH, NAFLD, etc.), drugs targeting *de novo* lipogenesis may be useful both as chemopreventive and therapeutic agents for liver cancer. Since inhibitors of FASN are already commercially available and used for the treatment of obesity with a good safety profile^36^, clinical trials using these drugs in liver cancer should be designed.

Finally, we showed that preneoplastic and neoplastic lesions from AKT/c-Met mice exhibit high levels of AKT/mTOR and Ras/MAPK cascades. In a previous study, we found the coordinated activation of AKT/mTOR and Ras/MAPK cascades in a subset of human HCCs with aggressive biological behavior^23^. Thus, the AKT/c-Met mouse model of hepatocarcinogenesis might represent a valid preclinical tool to investigate the therapeutic potential of various targeted therapies against HCC.

## Materials and Methods

### Constructs and reagents

The constructs used for mouse injection, including pT3-EF1α, pT3-EF1α-HA-myr-AKT, pT3-EF1α-V5-c-Met, pCMV-Cre and pCMV/sleeping beauty transposase (SB), were described previously^19^,^20^,^22^,^23^. miR-30 based shRNA against Raptor (shRaptor) and Renilla Luciferase (shLuc) were inserted into pT3-EF1α-HA-myr-AKT plasmid via the Gateway PCR cloning strategy (Invitrogen, Carlsbad, CA). Plasmids were purified using the Endotoxin free Maxi prep kit (Sigma-Aldrich, St.Louis, MO) before being injected into the mice.

### Hydrodynamic transfection and mouse monitoring

Wild-type FVB/N mice were obtained from Charles River (Wilmington, MA). The *FASN*^*fl/fl*^ mouse (in the C57BL/6 background) has been previously described^37^. *AlbCre* mice^38^ were purchased from Jackson Laboratory (Bar Harbor, ME). *AlbCre* mice were crossed with *FASN*^*fl/fl*^ mice to eventually generate liver specific *FASN* knockout mice (*AlbCre;FASN*^*fl/fl*^ mice). Hydrodynamic transfection was performed as described^19^,^20^,^22^,^23^,^39^,^40^. In brief, the plasmids encoding the gene(s) of interest along with sleeping beauty transposase (SB) in a ratio of 25:1 were diluted in 2 ml saline (0.9% NaCl), filtered through 0.22 μm filter, and injected into the lateral tail vein of the mice in 5 to 7 seconds. For high fat diet treatment of mice, AKT/c-Met/Cre injected *FASN*^*fl/fl*^ mice were fed high fat soft pellets with 60% fat calories (Bio‐Serv, Flemington, NJ) starting from the second day after the hydrodynamic injection for 10 weeks. Mice were housed, fed, and monitored in accordance with protocols approved by the Committee for Animal Research at the University of California, San Francisco.

### Immunohistochemical staining

Liver specimens were fixed in 4% paraformaldehyde and embedded in paraffin. Preneoplastic and neoplastic liver lesions were assessed by two board-certified pathologists (M.E. and F.D.) in accordance with the criteria by Frith *et al.*^41^, as previously described in detail^40^. For immunohistochemistry, deparaffinized sections were incubated in 3% H_2_O_2_ dissolved in 1× phosphate-buffered saline (PBS) for 30 minutes to quench the endogenous peroxidase. For antigen retrieval, slides were microwaved in 10 mM citrate buffer (pH 6.0) for 12 minutes. Subsequently, slides were incubated with primary antibodies (Supplementary Table 2) overnight at 4 °C. All the primary antibodies used in the present investigation were selected among those that were previously validated by the manufacturers for immunohistochemistry. The immunoreactivity was visualized with the Vectastain Elite ABC kit (Vector Laboratories, Burlingame, CA), using Vector NovaRED™ (Vector Laboratories) as the chromogen. Slides were counterstained with Mayer’s hematoxylin.

### Protein Extraction and Western blotting

Frozen mouse liver specimens were homogenized in Mammalian protein extraction reagent (Thermo Scientific, Waltham, MA) containing the Complete Protease Inhibitor Cocktail and sonicated. Protein concentrations were determined with the Bio-Rad Protein Assay Kit (Bio-Rad, Hercules, CA) using bovine serum albumin as standard. Aliquots of 40 μg lysate were denatured by boiling in Tris-Glycine SDS Sample Buffer (Invitrogen), separated by SDS-PAGE, and then transferred onto nitrocellulose membranes (Invitrogen, Grand Island, NY). Membranes were blocked in 5% non-fat dry milk in Tris-buffered saline containing 0.1% Tween 20 for 1 hour and probed with specific antibodies listed in Supplementary Table 2. Each primary antibody was followed by incubation with horseradish peroxidase-secondary antibody diluted 1:10,000 for 1 hour and then revealed with the SuperSignal West Pico Chemiluminescent Substrate (Pierce Chemical Co., New York, NY). Equal loading was assessed by Ponceau Red reversible staining as well as GAPDH and β-Actin Western blotting.

### Quantitative reverse transcription real-time polymerase chain reaction (qRT-PCR)

Validated Gene Expression Assays for human *FASN* (ID: Hs01005622_m1), *c-Met* (ID: Hs01565584_m1), and *β-Actin* (ID: 4333762T) were purchased from Applied Biosystems (Foqter City, CA). PCR reactions were performed with 100 ng of cDNA from HepG2 and HLF cell lines, using the RotorQ (Qiagen, Valencia, CA) thermal cycler and the TaqMan Universal PCR Master Mix (Applied Biosystems). Cycling conditions were: 10 min of denaturation at 95 °C and 40 cycles at 95 °C for 15 s and at 60 °C for 1 min. Quantitative values were calculated by using the PE Biosystems Analysis software (Applied Biosystems) and expressed as N target (NT). NT = 2-^ΔCt^, wherein the ΔCt value of each sample was calculated by subtracting the average Ct value of the target gene from the average Ct value of the *β-Actin* gene.

### *In vitro* experiments

The HLF, HepG2, and HLE human hepatoma cell lines were used for the *in vitro* studies. Cell lines were maintained as monolayer cultures in Dulbecco’s modified Eagle medium supplemented with 10% fetal bovine serum. For knockdown studies, HLF and HepG2 cells were transfected with 50 pmol of scramble small interfering RNA (siRNA) or siRNA directed against human FASN (ID # s5032) gene from Life Technologies (Grand Island, NY), according to the manufacturer’s recommendations, and incubated for 48 hours. For the treatment with chemical inhibitors, the three cell lines were plated at 2.0 × 10^3^/well in 96-well plate and grown for 12 hours. After 24-hour serum deprivation, the vehicle (DMSO; Sigma-Aldrich), C75 (Cayman Chemical, Ann Arbor, MI; 100 μmol/L), Cycloheximide (Santa Cruz Biotechnology, Santa Cruz, CA; 53, 3 μmol/L), 4EGI-1 (EMD Millipore, Billerica, MA; 100 μmol/L), MG132 (Sigma-Aldrich; 20 μmol/L), EMD1214063 (Selleck Chemicals, Houston, TX; 5 μmol/L) and/or MK2206 (Santa Cruz Biotechnology; 5 μmol/L) were added to the medium and cells incubated for 24 and 48 hours.To assess cell proliferation, the three cell lines were plated at the concentration of 2.0 × 10^3^/well in 96-well plates, allowed to attach and adjust for the next 12 and grown for additional 48 hours. The proliferation was assessed at 48 hours with the BrdU Cell Proliferation Assay Kit (Cell Signaling Technology, Danvers, MA) by measuring the absorbance at 450 nm following the manufacturer’s protocol. To measure apoptosis, cell lines were plated at the concentration of 2.0 × 10^3^/well in 96-well plates, incubated for 12 hours, and then subjected to 24-hour serum deprivation. Cell lines continued to grow in serum-free medium for additional 48 hours. Apoptosis was assessed at the latter time point with the Cell Death Detection Elisa Plus Kit (Roche Molecular Biochemicals, Indianapolis, IN) by measuring the absorbance at 415 nm, following the manufacturer’s instructions. All cell line experiments were repeated at least three times in triplicate.

### Human Liver Tissue Specimens

A collection of 94 formalin-fixed, paraffin-embedded HCC samples was used in the present study. HCC specimens were either kindly provided by Dr. Snorri Thorgeirsson (National Cancer Institute, Bethesda, MD, USA) or collected at the Institute of Pathology of the University of Greifswald (Greifswald, Germany). The clinicopathological features of the patients are reported in Supplementary Table 1. Institutional Review Board approval was obtained at the National Institutes of Health and the University of Greifswald. For use of patient tissues, protocol approval was obtained by the Ethics Review Board of Greifswald University (Greifswald, Germany). Informed consent was obtained from all subjects. Investigation has been conducted in accordance with the ethical standards and according to the Declaration of Helsinki as well as according to national and international guidelines.

### Statistical analysis

Data analysis was performed with Prism 6 (GraphPad, San Diego, CA). All data are presented as Means ± SE. Comparisons between two groups were performed with two-tailed unpaired *t* test. Comparisons between three or more groups were performed with ANOVA. *P* values < 0.05 were considered statistically significant.

The experimental methods were carried out in accordance with the approved guidelines.

## Additional Information

**How to cite this article**: Hu, J. *et al.* Co-activation of AKT and c-Met triggers rapid hepatocellular carcinoma development via mTORC1/FASN pathway in mice. *Sci. Rep.* **6**, 20484; doi: 10.1038/srep20484 (2016).

## Supplementary Material

Supplementary Information
