# Supplementary material for: Co-activation of AKT and c-Met triggers rapid hepatocellular carcinoma development via the mTORC1/FASN pathway in mice
Source: Sci Rep. 2016 Feb 9;6:20484. doi: 10.1038/srep20484 (PMC4746674; doi:10.1038/srep20484)
Supplement: Supplementary Information [file srep20484-s1.pdf]

Co-activation of AKT and c-Met triggers rapid hepatocellular carcinoma development via the mTORC1/FASN pathway in mice

Junjie Hu[1,2], Li Che[2,3], Lei Li[2,4], Maria G. Pilo[5], Antonio Cigliano[6], Silvia Ribback[6], Xiaolei Li[2,7], Gavinella Latte[5], Marta Mela[5], Matthias Evert[8], Frank Dombrowski[6], Guohua Zheng[1,*], Xin Chen[1,2,*], and Diego F. Calvisi[5,*]

[1]School of Pharmacy, Hubei University of Chinese Medicine, Wuhan, Hubei, P.R. China

[2]Department of Bioengineering and Therapeutic Sciences and Liver Center, University of California, San Francisco, CA, USA

[3]Key Laboratory of Carcinogenesis and Translational Research (Ministry of Education), Peking University Cancer Hospital and Institute, Beijing, P. R. China

[4]School of Pharmacy, Tongji Medical College, Huazhong University of Science and Technology, Wuhan, Hubei, P. R. China

[5]Department of Clinical and Experimental Medicine, University of Sassari, Sassari, Italy

[6]Institute of Pathology, University of Greifswald, Greifswald, Germany

[7]Department of Hepatobiliary Surgery, Xijing Hospital, The Fourth Military Medical University, Xi'an, Shaanxi, P.R. China

[8]Institute of Pathology, University of Regensburg, Regensburg, Germany

Junjie Hu, Li Che and Lei Li contributed equally to the work.

***Corresponding authors:** Diego F. Calvisi, Department of Clinical and Experimental Medicine, University of Sassari, via Padre Manzella 4, 07100 Sassari, Italy. Tel: 0039 079 228306; Fax: 0039 079 228305; e-mail: calvisid@uniss.it; Xin Chen, UCSF, 513 Parnassus Ave., San Francisco, CA 94143, U.S.A. Tel: (415) 502-6526; Fax: (415) 502-4322; e-mail: xin.chen@ucsf.edu; or Guohua Zheng, Hubei University of Chinese Medicine, Wuhan, 430065 Hubei, P.R. China. Tel: 8602768890113; e-mail: zgh1227@sina.com.

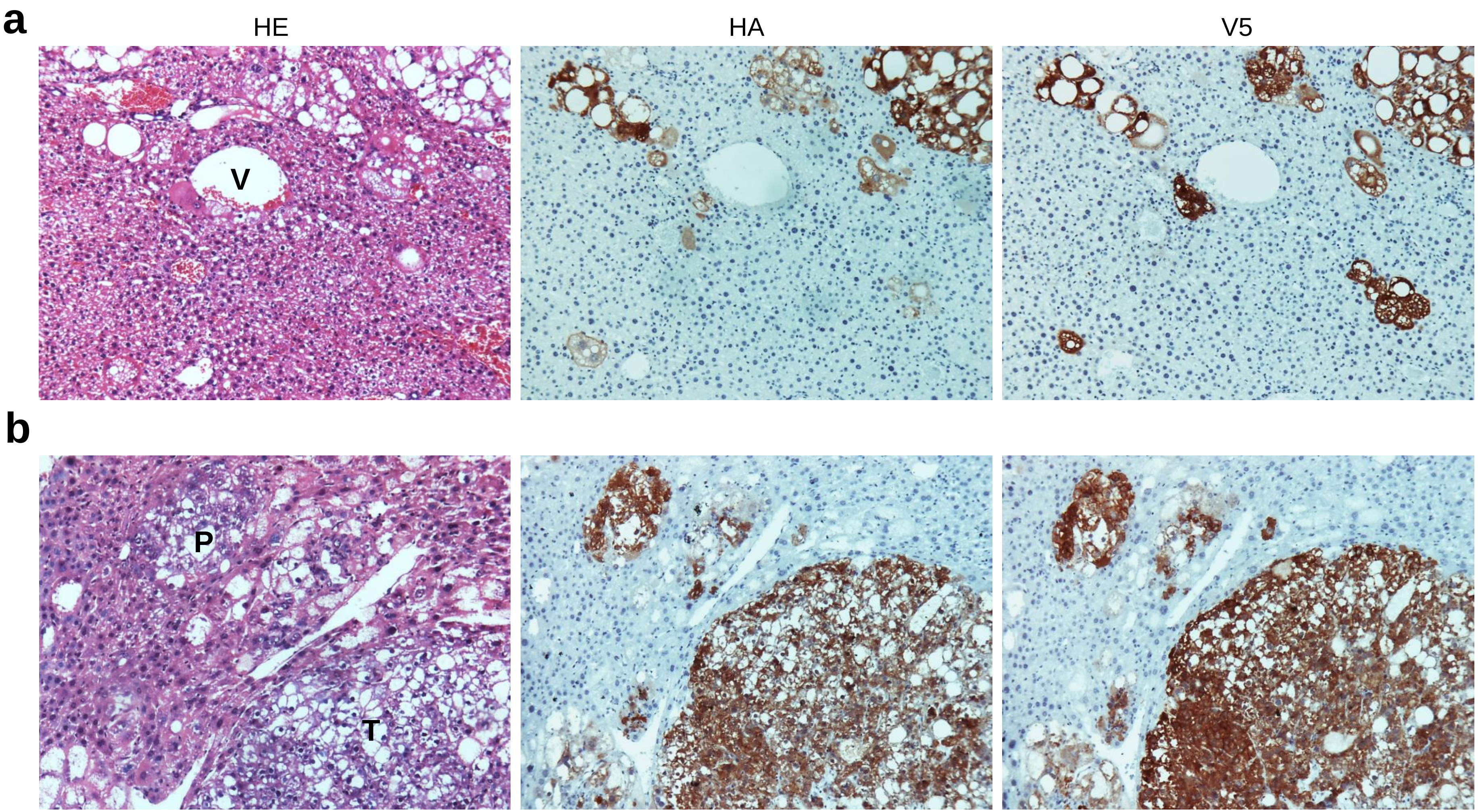

Supplementary Figure 1. Co-localization of HA-tagged AKT and V5-tagged c-Met immunoreactivity in preneoplastic and neoplastic liver lesions developed in AKT/c-Met mice. (**a**) Preneoplastic liver lesions, located within zone 3 of the liver acinus in the proximity of the hepatic venule (V), that consist of clusters of lipid-rich hepatocytes display immunoreactivity for both HA and V5 tags. (**b**) A clear-cell hepatocellular tumor (T) and a preneoplastic lesion (P) exhibiting co-localization of HA and V5 immunostaining. Original magnification: 100X. Abbreviation: HE, hematoxylin and eosin staining.

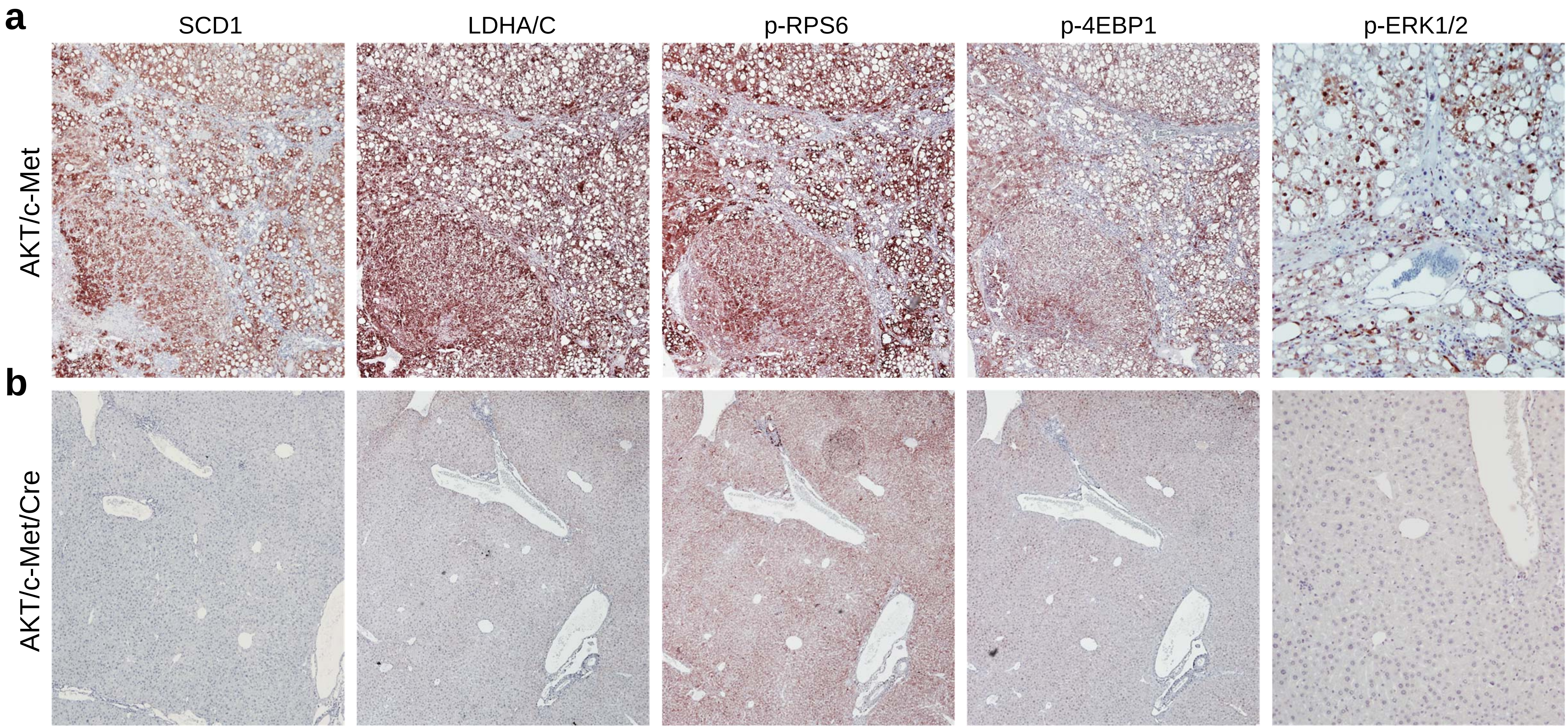

Supplementary Figure 2. AKT1/c-Met hepatocarcinogenesis is abolished by FASN depletion in mice. (**a**) Co-expression of AKT and c-Met proto-oncogenes in *FASN*$^{fl/fl}$ mice retaining an intact *FASN* gene (indicated as AKT/c-Met) triggered rapid hepatocarcinogenesis, which was accompanied by elevated levels of markers of lipogenesis (SCD1), glycolysis (LDHA/C), and mTORC1 activation (p-RPS6 and p-4EBP1). (**b**) Of note, Cre-mediated depletion of *FASN* gene in these mice (indicated as AKT/c-Met/Cre) completely suppressed tumor development and immunolabeling for the same proteins. Original magnification: 100X for p-ERK1/2; 40X for the others pictures.

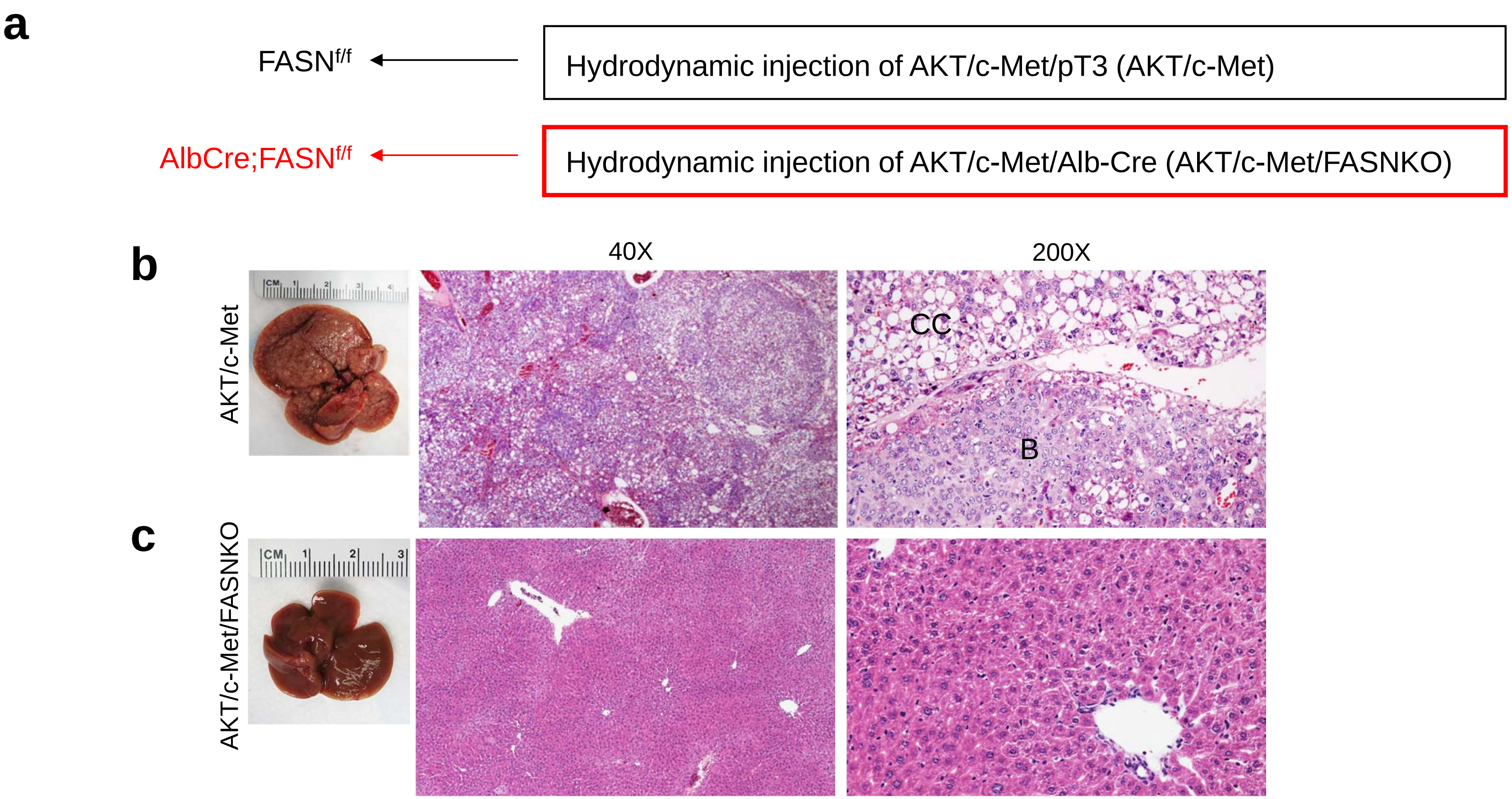

Supplementary Figure 3. Genetic ablation of FASN in the mouse liver suppresses tumor development driven by *AKT* and *c-Met* co-expression. (**a**) Study design. (**b**) Overexpression of *myr-AKT1* and *c-Met* led to lethal burden of liver tumor by 8 weeks post hydrodynamic injection in *FASN*$^{fl/fl}$ mice with an intact FASN gene (AKT/c-Met mice). AKT/c-Met livers were pale, enlarged, with numerous nodules covering the organ surface. Microscopically, AKT/c-Met livers were occupied by numerous hepatocellular tumors with either clear cell (CC) or basophilic (B) phenotype. (**c**) In striking contrast, AlbCre-mediated depletion of *FASN* gene in *FASN*$^{fl/fl}$ mice injected with *myr-AKT1* and *c-Met* (indicated as AKT/c-Met/FASNKO mice) completely abolished tumor development. Livers of AKT/c-Met/FASNKO mice were indeed completely normal 20 weeks post hydrodynamic injection. Original magnifications: 40X and 200X.

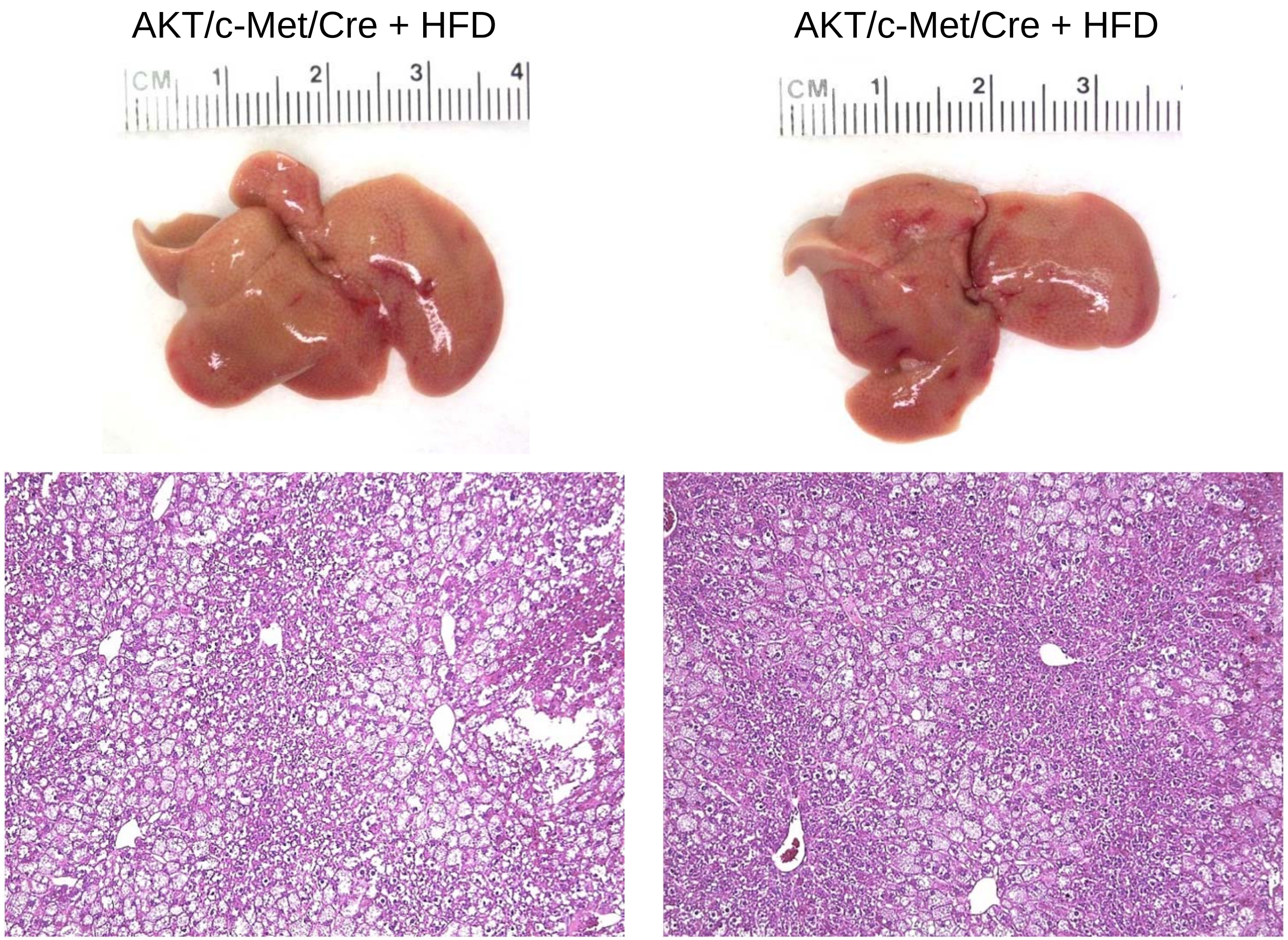

Supplementary Figure 4. Exogenous supplementation of lipids does not compensate the loss of FASN in AKT/c-Met mice. AKT/c-Met/Cre injected *FASN*$^{fl/fl}$ mice (indicates as AKT/c-Met/Cre) were fed a high fat diet (HFD) for 10 weeks. Livers of these mice appeared macroscopically pale (upper panels) and exhibited extensive steatosis at the histological level (lower panels), but did not show any sign of malignant transformation. Two examples are shown. Original magnification: 40X.

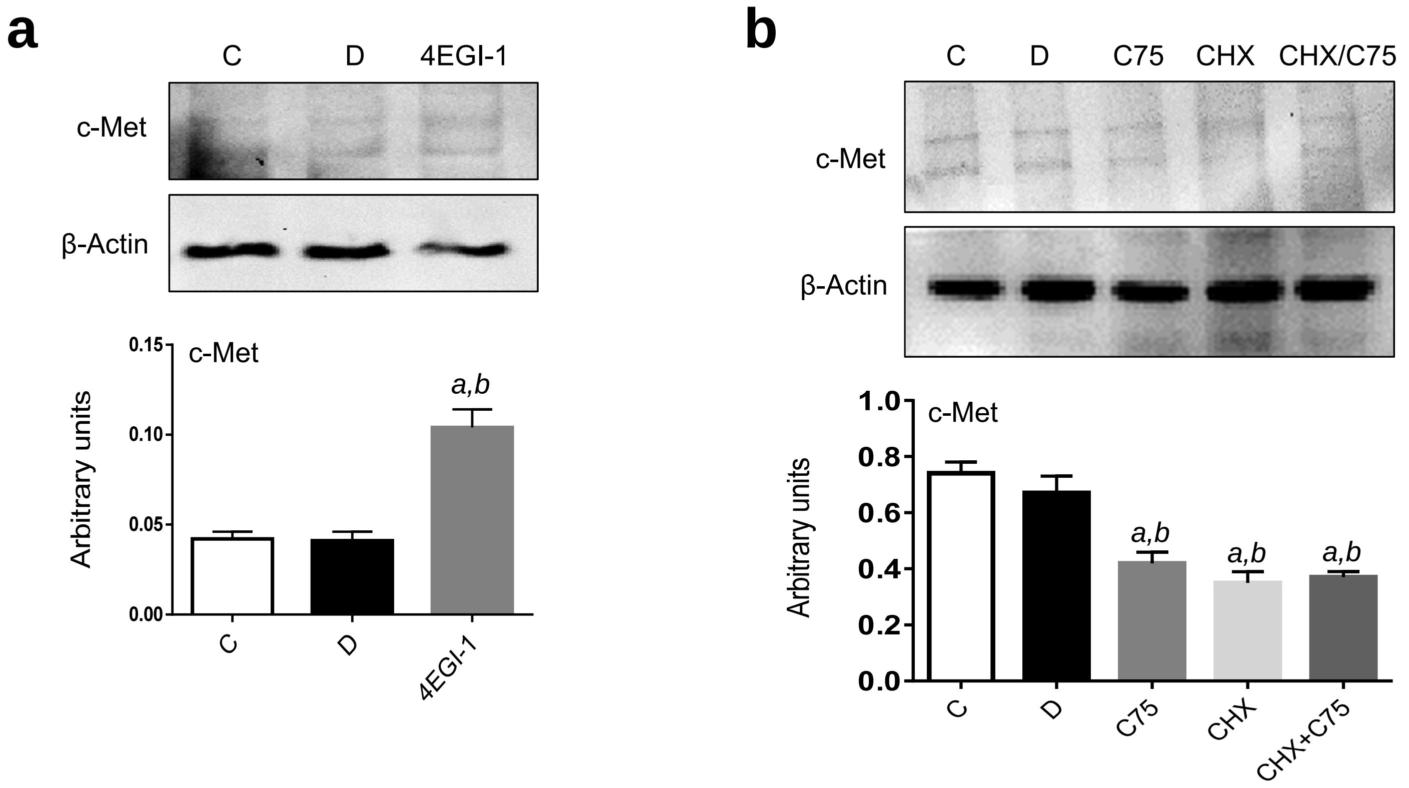

Supplementary Figure 5. Protein stability but not cap-dependent translation regulates c-Met levels in the HepG2 hepatoma cell line. (**a**) Treatment with the cap-dependent translation inhibitor, 4EGI-1 (100μM), induces rather than downregulates the levels of c-Met. Upper panel: Western blot analysis. Lower panel: densitometric analysis of protein bands, after normalization to β-Actin levels. (**b**) Treatment with the protein synthesis inhibitor, cycloheximide (CHX), induces downregulation of c-Met protein levels, similar to that obtained by administration of the FASN inhibitor, C75 (100μM). Of note, the two treatments do no induce a synergistic effect in reducing c-Met levels. Upper panel: Western blot analysis. Lower panel: densitometric analysis of protein bands, after normalization to β-Actin levels. Optical densities of the peaks were calculated using the Quantity One software (Bio-Rad, Hercules, CA), normalized to β-actin levels, and expressed in arbitrary units. Each bar represent mean ± SD of three independent experiments conducted in triplicate. The forty-eight hour time point of the treatment with 4EGI-1, C75, CHX, and CHX+75 is shown; no differences were seen in each treatment vs. control 24h after treatment (not shown). Tukey-Kramer's test: *P* at least <0.001; *a*, versus control (C); *b*, versus DMSO (D).

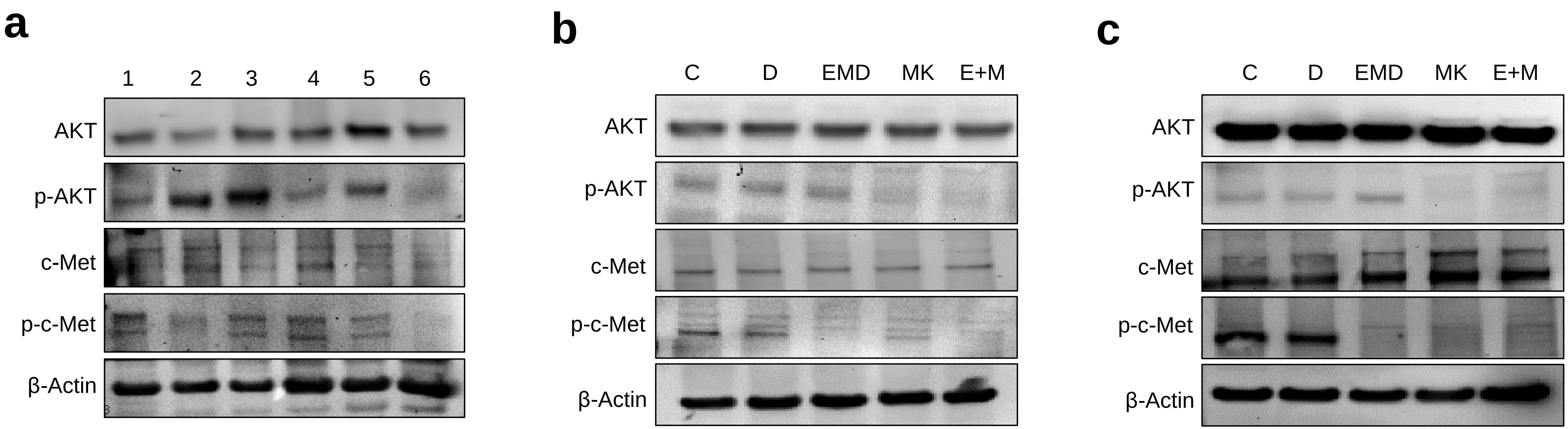

Supplementary Figure 6. Suppression of AKT triggers downregulation of c-Met activation in HLE and HLF human HCC cell lines. (**a**) Protein levels of total AKT (AKT), phosphorylated/activated AKT (p-AKT), total c-Met (c-Met), and phosphorylated/activated c-Met (p-c-Met) in HuH6 (1), HuH7 (2), HLE (3), SKP-Hep1 (4), HLF (5), and HepG2 (6) cell lines as detected by Western blot analysis. (**b**) Treatment with the AKT inhibitor, MK2206 (MK; 5µmol/L), c-Met inhibitor, EMD1214063 (EMD; 5µmol/L), or the combination of the two inhibitors (E+M) was performed on the HLE cell line. As expected, activated/phosphorylated AKT and c-Met protein levels were downregulated following treatment with MK2206 and EMD1214063, respectively). Of note, treatment with the AKT inhibitor also resulted in a striking decrease of activated c-Met levels. Equivalent results were obtained in HLF cells subjected to the same treatments (**c**). β-Actin was used as a loading control.

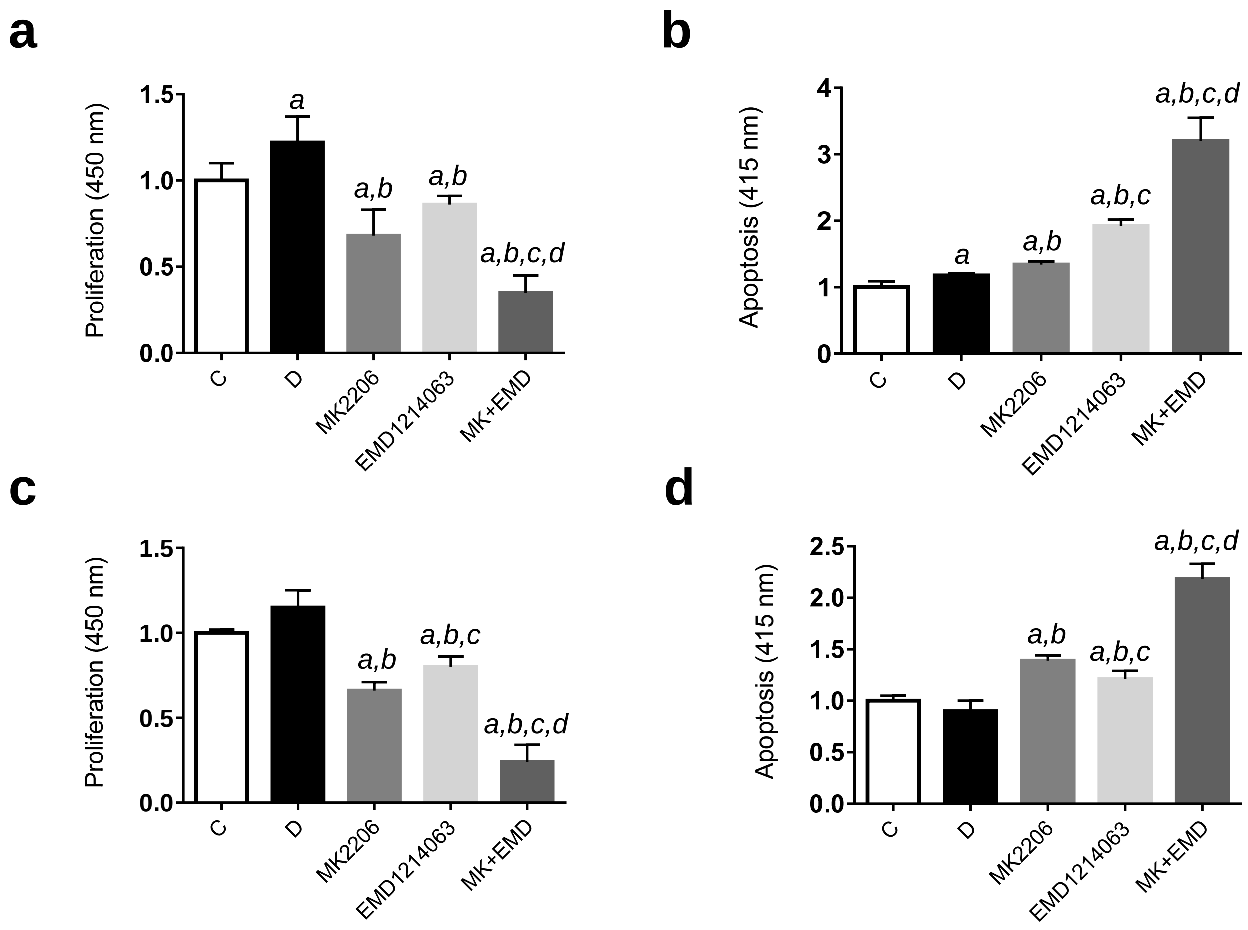

Supplementary Figure 7. Suppression of AKT and c-Met activity via specific inhibitors is highly detrimental for the growth of human HLE and HLF hepatocellular carcinoma (HCC) cell lines *in vitro*. (**a**,**b**) Treatment with the AKT inhibitor, MK2206 (MK; 5μM/L), or the c-Met inhibitor, EMD1214063 (EMD; 5μM/L), decreased proliferation (**a**) and induced apoptosis (**b**) in the HLE HCC cell line when compared with control (C; untreated) and DMSO (D; solvent) treated cells. Of note, combined administration of MK2206 and EMD1214063 (MK+EMD) further decreased the proliferation rate and increased the apoptosis degree of HLE cells (**c**,**d**). A similar growth restraint pattern to that described in HLE cells was also detected in HLF cells when subjected to the administration of the two inhibitors, either alone or in combination. Each bar represent mean ± SD of three independent experiments conducted in triplicate. Tukey-Kramer's test: P at least < 0.05; *a*, versus control (untreated cells); *b*, versus DMSO (solvent); *c,* versus MK2206; *d*, versus EMD1214063.

Supplementary Table 1. Clinicopathological features of HCC Patients

Variables	Features	
	HCCB[a]	HCCP[b]
No. of patients	44	50
Male	29	36
Female	15	14
Age (Mean ± SD)	62.2 ± 10.6	66.4 ± 12.4
Etiology		
HBV	20	24
HCV	18	18
Ethanol	4	6
NA	2	2
Cirrhosis		
+	34	42
-	10	8
Tumor size		
> 5 cm	28	31
< 5 cm	16	19
Edmondson and Steiner grade		
II	12	10
III	20	24
IV	12	16
Alpha-fetoprotein secretion		
> 300 ng/ml of serum	26	28
< 300 ng/ml of serum	18	26
Survival after partial liver resection (months) Means ± SD	60.2 ± 19.8	21.8 ± 9.5

[a]HCCB, HCC with better outcome/longer survival (survival >3 years following partial liver resection)

[b]HCCP, HCC with poorer outcome/shorter survival (survival <3 years following partial liver resection)

Supplementary Table 2. List of the primary antibodies used for Western blot analysis (WB) and/or immunohistochemistry (IHC)

Protein	Antibody (and catalog number)	Epitope mapping (and application)
Phospho-AKT	Rabbit monoclonal (4060)	Serine 473/472† (WB; IHC)
Phospho-AKT	Rabbit monoclonal (13038)	Threonine 308† (WB; IHC)
t-AKT	Rabbit monoclonal (4691)	COOH-terminus† (WB)
V5-Tag	Mouse monoclonal (sc-81594)	V5-Tag* (WB; IHC)
HA-Tag	Mouse monoclonal (2367)	HA-Tag† (WB; IHC)
SCD1	Rabbit monoclonal (2794)	Residues around Leu35† (WB; IHC)
FASN	Mouse monoclonal (610962)	Amino acids 9-202* (WB; IHC)
Phospho-4EBP1	Rabbit monoclonal (2855)	Thr37/46† (WB;IHC)
Phospho-RPS6	Rabbit monoclonal (5364)	Ser240/244† (WB; IHC)
PKM1	Rabbit monoclonal (7067)	Residues around Ala411† (WB)
PKM2	Rabbit monoclonal (4053)	Full length† (WB)
LDHA/C	Rabbit monoclonal (3558)	Full length† (WB; IHC)
Raptor	Rabbit monoclonal (2280)	Full length† (WB)
c-Met	Rabbit monoclonal (8198)	NH2-terminus† (WB; IHC)
Phospho-Met	Rabbit monoclonal (3129)	Tyr 1234/1235† (WB)
t-ERK1/2	Rabbit monoclonal (9102)	C- terminus† (WB; IHC)
Phospho-ERK1/2	Rabbit monoclonal (4370)	Thr202/Tyr204† (WB; IHC)
Phospho-mTOR	Rabbit polyclonal (2971)	Ser2448† (WB)
β-Actin	Mouse monoclonal (A1978)	Full length* (WB)
GAPDH	Rabbit polyclonal (sc-25778)	Full length* (WB; IHC)

† Provided by Cell Signaling Technology Inc. (Danvers, MA).

* Provided by Santa Cruz Biotechnology (Santa Cruz, CA).
